# Electroacupuncture of Guanyuan (CV4) Acupoint Improved Pelvic Inflammatory Disease Pain by Inhibiting Neuroinflammation and Sympathetic Activity

**DOI:** 10.1002/iid3.70299

**Published:** 2025-11-05

**Authors:** Jinyu Qu, Yingchun Peng, Xuefang Shen, Jin Xiong, Huan Wang, Xiang Xiao, Yili Wang

**Affiliations:** ^1^ Department of Neurology the First Affiliated Hospital of Chengdu Medical College Chengdu China; ^2^ Department of Rehabilitation Medicine the First Affiliated Hospital of Chengdu Medical College Chengdu China; ^3^ Department of Nephrology First Affiliated Hospital of Chengdu Medical College Chengdu China

**Keywords:** electroacupuncture, Guanyuan acupoint, neuroinflammation, pelvic inflammatory disease, sympathetic activity

## Abstract

**Objective:**

To investigate the underlying mechanisms through which electroacupuncture (EA) at the Guanyuan (CV4) acupoint inhibits sympathetic activity and neurogenic inflammatory responses to relieve pain in rats with pelvic inflammatory disease (PID).

**Methods:**

*Escherichia coli* and *Staphylococcus aureus* were used to establish a PID rat model. EA was evaluated at frequencies of 2, 100, and 2/100 Hz, and 2/100 Hz was selected for subsequent investigation. The rats were randomly divided into the control, model, EA‐guanyuan (2/100 Hz), and EA‐nonsensitized groups (*n* = 6). Mechanical withdrawal threshold (MWT) and thermal withdrawal latency (TWL) were assessed using von Frey filaments. Hematoxylin and eosin staining was performed to evaluate the histopathology. The tyrosine hydroxylase (TH) expression was analyzed using immunofluorescence (IF) staining. The levels of tumor necrosis factor‐α (TNF‐α), interleukin‐2 (IL‐2), transforming growth factor‐β1 (TGF‐β1), intercellular cell adhesion molecule‐1 (ICAM‐1), 5‐hydroxytryptamine receptor 3 (5‐HT3R), substance P (SP), hyaluronic acid (HA), and bradykinin (BK) were measured using an enzyme‐linked immunosorbent assay (ELISA). Western blot analysis was performed to measure the expression of 5‐HT3R, calcitonin gene‐related peptide (CGRP), HA, Kininogen 1 (KNG1), prostaglandin I2 (PGI2), and trefoil factor 2 (TFF2). Transmission electron microscopy (TEM) was used to observe synaptic connections.

**Results:**

EA at CV4 reduced the behavioral pain score (*p* < 0.05), increased MWT and TWL, and alleviated uterine tissue pathological damage in rats. EA at CV4 reduced the levels of 5‐HT3R, CGRP, BK, HA, KNG1, PGI2SP, TGF‐β1, ICAM‐1, and TNF‐α, and increased IL‐2 levels (*p* < 0.05). Furthermore, EA at CV4 inhibited sympathetic activity by decreasing TH expression (*p* < 0.05). Additionally, EA at CV4 restored the synaptic connections between the pelvic nerves of the dorsal commissural neuron (DCN).

**Conclusion:**

EA at CV4 alleviated the pathological damage and pain sensitization of uterine tissue in rats with PID by inhibiting sympathetic activity and neurogenic inflammatory response.

## Graphical abstract

Electroacupuncture at Guanyuan (CV4) acupoint reduced the behavioral pain score and alleviated the pathological damage and pain sensitization of uterine tissue in PID rats by inhibiting sympathetic activity and neurogenic inflammatory response.

**Abbreviations**
5‐HT3R: 5‐hydroxytryptamine receptor 3
BK: bradykinin
CGRP: calcitonin‐gene‐related peptide
CV4: Guanyuan
DCN: dorsal commissural neuron
EA: electroacupuncture
HA: hyaluronic acid
ICAM‐1: intercellular cell adhesion molecule‐1
IL‐2: interleukin‐2
KNG1: Kininogen 1
MWT: Mechanical withdrawal threshold
PBN: parabrachial nucleus.
PGI2: prostaglandin I2
PID: pelvic inflammatory disease
SP: substance P
TCM: traditional Chinese medicine
TFF2: trefoil factor 2
TGF‐β1: transforming growth factor‐β1
TNF‐α: tumor necrosis factor‐α
TWL: thermal withdrawal latency

## 1 Introduction

One of the most prevalent conditions affecting women is pelvic inflammatory disease (PID), an inflammatory condition that includes endometritis, salpingitis, tubo‐ovarian abscess, and pelvic peritonitis [1, 2]. The clinical symptoms of inflammation primarily manifest as lower abdominal pain, which seriously affects the patient's quality of life and physical health. Regarding the pathogenesis of PID, studies generally believe that it is primarily caused by infection with pathogenic microorganisms, including *Chlamydia trachomatis*, *Mycoplasma genitalium*, *Neisseria gonorrhoeae*, and *Escherichia coli* [3]. Studies also have shown that *Staphylococcus aureus*, *Streptococcus pyogenes*, *Streptococcus agalactiae*, *Escherichia coli*, and *Neisseria gonorrhoeae* are potential vaginal pathogens [4]. When the pelvic organs are infected by pathogens, it triggers an inflammatory response and releases inflammatory mediators, which stimulate nerve endings and cause pain [5, 6]. Therefore, PID treatment typically involves the use of antibiotics, such as penicillin and cephalosporins [7]. However, long‐term use of antibiotics can easily lead to drug resistance and reduced efficacy owing to inflammatory infiltration, fibrous tissue, and hoof tissue adhesions [8]. Therefore, it is imperative to develop safer and more efficient treatments.

In recent years, traditional Chinese medicine (TCM) has conducted in‐depth research on the treatment of PID [9, 10]. According to TCM theory, PID belongs to the syndrome of heat invading blood chamber, an abnormal increase of leucorrhea, menstrual abdominal pain, and infertility, according to the famous classic book named “Synopsis of the Golden Chamber.” Furthermore, it is worth noting that acupuncture plays an important role in the treatment of PID. Research has shown that acupuncture treatment for PID has significant therapeutic effects and can help improve the symptoms of pelvic inflammation and alleviate pain in patients [11]. According to TCM theory, Wu et al. [12] studied the relationship between referred pain and acupuncture sensitization caused by pelvic visceral diseases. According to the schematic diagram of the distribution segments of human dermatomes [13], the sensory input of the pelvic organs belongs to the T10‐S1 segment. Patients with gynecological diseases, such as PID, ovarian cysts, and ectopic pregnancy, primarily experience referred pain in the lower abdomen, an area rich in common meridians and acupuncture points for treating gynecological diseases, primarily focusing on the T10‐S1 segment [12]. Some areas of referred pain overlap with the locations of acupoints such as the Ren Meridian, the Liver Meridian of the Foot‐Jueyin, the Kidney Meridian of the Foot‐Shaoyin, and the Spleen Meridian of Foot‐Taiyin, which are key acupoints for the treatment of gynecological diseases. Research has reported that acupuncture at Zhongji (CV3), Guanyuan (CV4), Zigong (EX‐CA1), Zusanli (ST36), and Sanyinjiao (SP6) acupoints reduced TNF‐α and IL‐10 [10]. Notably, Guanyuan acupoint (CV4) belongs to the Ren Meridian, located in the lower abdomen and adjacent to the uterus, and is an important acupoint for the treatment of diseases of the reproductive system. The Guanyuan acupoint is a key acupuncture point used for the treatment of acute and chronic gynecological diseases. Acupointure at CV4 can invigorate the kidney, replenish essence, promote blood circulation, and nourish the qi essence to help the congenital foundation. Clinical evidence has shown that acupuncture at CV4 can improve symptoms in patients with chronic PID, including lumbosacral and lower abdominal pain [14]. Acupuncture at CV4 could effectively improve pelvic blood circulation and promote the dissipation and absorption of pelvic inflammatory secretions [15]. In addition, acupuncture at CV4 could produce anti‐inflammatory and analgesic effects by inhibiting the P2X7R/NLRP3 signaling pathway and suppressing the release of inflammatory cytokines in rats with chronic pelvic pain syndrome (CPPS) [16]. This suggests that acupuncture at CV4 may be an effective treatment for PID. Therefore, the Guanyuan point, located in the lower abdomen, was chosen as the sensitized acupuncture point for PID.

Although several research confirming a strong link between pain sensitivity and pain perception, a complete understanding of the pathophysiological mechanisms underlying pain remains elusive. The process by which acupoints go from a “silent” to a “sensitized” state in pathological circumstances is known as “acupoint sensitization”. Visceral pathologies can cause secondary somatic pain and induce sensitization of acupoints, whereas stimulating these sensitized points can ameliorate visceral function and relieve secondary somatic pain. Our previous research has shown that electroacupuncture (EA) stimulation of sensitized acupoints (Zusanli) can reduce sympathetic‐sensory coupling structures and neurogenic inflammatory responses, thereby relieving somatic pain in rats with colitis [17, 18, 19]. However, the precise mechanism through which pain sensitivity contributes to the improvement in PID through EA at the Guanyuan acupoint remains unclear.

Therefore, this study examined the potential of EA at CV4 to suppress sensitized acupoints and neurogenic inflammatory responses. In addition, we examined the molecular mechanism through which EA stimulation of the CV4 alleviates pain in rats with PID, using a pathogenic bacteria‐induced PID rat model.

## 2 Materials and Methods

### 2.1 Animals

Chengdu Dashuo Biotechnology Co. Ltd. (Sichuan, China) provided sixty female Sprague‐Dawley (SD) rats (body weight, 180–200 g). The rats were kept under standard pathogen‐free conditions for 1 week to acclimatize them to the environment. During the experimental period, the rats were allowed to drink freely, while maintaining a normal 12 h light/12 h dark cycle. All experiments were approved by the Experimental Animal Welfare Ethics Committee of the Chengdu Medical College (Approval No. 2023012).

### 2.2 Effects of EA at Guanyuan Acupoint at Different Frequencies on the Pain Behavior of Rats

**Figure 1:**
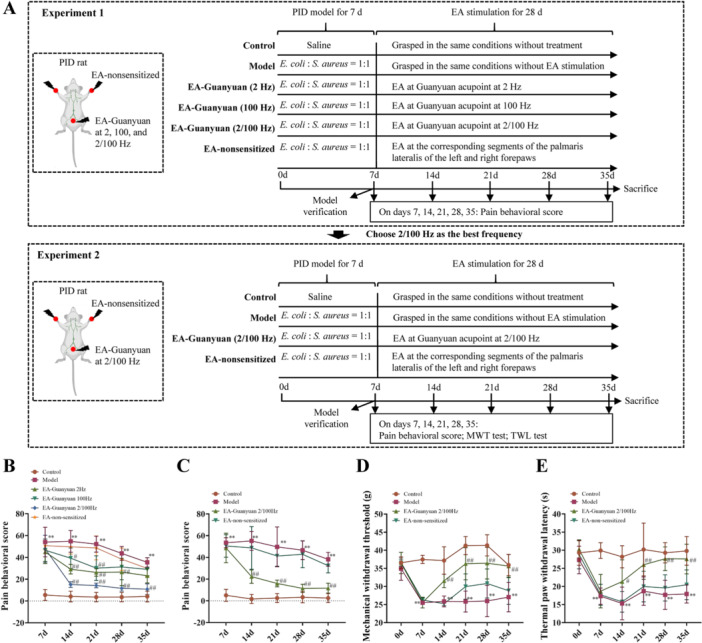
Effects of EA stimulation of Guanyuan acupoint on pain behavior of rats with PID. (A) Schematic diagram of the experimental procedure in this study. (B) Behavioral pain score of rats on days 7, 14, 21, 28, and 35 after EA stimulation of Guanyuan acupoint at different frequencies at 2, 100, and 2/100 Hz. (C) Behavioral pain score of rats on days 7, 14, 21, 28, and 35 after EA stimulation of Guanyuan acupoint at 2/100 Hz. Mechanical and thermal hypersensitivity in rats with PID was assessed by measuring the MWT (D) and TWL (E). Values are expressed as mean ± SD (*n* = 6), **p < 0.01, versus control group; ^#^ *p* < 0.05, ^##^ *p* < 0.01, versus model group. EA, electroacupuncture; PID, pelvic inflammatory disease; MWT, mechanical withdrawal threshold; TWL, thermal withdrawal latency.

The rats were divided into six groups at random: the control, model, EA‐Guanyuan (2 Hz), EA‐Guanyuan (100 Hz), EA‐Guanyuan (2/100 Hz), and EA‐non‐sensitized groups (*n* = 6 per group). A PID rat model was established as previously described [20, 21]. Briefly, *E. coli* and *S. aureus* were diluted 1:1 in sterile saline to form a mixture with a concentration of approximately 1 × 10^9^/mL. Rats were anesthetized with isoflurane and prepared for surgery by shaving and disinfecting the abdominal area. The abdominal cavity was opened using ophthalmic surgical instruments, and the uterine tubes were exposed. A syringe was then gently inserted into the uterine cavity along the direction of the tube. The model group was injected with 0.05 mL of bacterial suspension, whereas the control group received 0.05 mL of saline. After 7 days of establishing the model, the pain behavioral responses of the rats were observed for 10 min. The rats were then slightly immobilized without significantly inhibiting their activity. Two sterile stainless‐steel needles with a diameter of 0.35 mm connected to the HANS nerve stimulator LH202H were inserted into the Guanyuan acupoint. The location of Guanyuan acupoint was determined according to the “acupoint atlas of Xingbang Hua”, which was located 25 mm below the belly button of rats [22]. Three different frequencies of EA stimulation were applied to the rats at 2, 100, and 2/100 Hz, with 1, 2, and 3 mA for 5 min each, for a total of 15 min, the EA frequencies refer to a previously reported method [23]. EA non‐sensitized rats were stimulated with the corresponding segments of the palmaris lateralis of the left and right forepaws (Figure 1A). EA treatment was performed from 9AM to 12AM once daily for 28 days. Measurements of pain behavioral scores were obtained from 9AM to 12AM on days 7, 14, 21, 28, and 35. PID pain primarily occurs in the lower abdomen, and the corresponding segments on the lateral side of the front paws of the rats do not belong to the reference area of PID pain. There is no direct neural connection between the nerve conduction pathway of the forepaw and T10‐S1; therefore, it can be used as a control point to exclude the effects of nonspecific or generalized pain. To confirm the accuracy of selecting the acupoints (CV4) in the tissue, non‐acupoints on both sides were selected for comparison. In addition, the selection of non‐sensitized acupoint areas can prevent direct stimulation of the lesion area (pelvic cavity) and reduce pain in animals.

### 2.3 Effects of EA at Guanyuan Acupoint on Chronic Pelvic Inflammation Rats

The rats were divided into four groups at random: control, model, EA‐Guanyuan (2/100 Hz), and EA‐nonsensitized groups (*n* = 6 per group). Only the control and model groups were moderately well‐understood. The EA‐Guanyuan group was stimulated with EA at the guanyuan acupoint. The non‐sensitized EA group was stimulated with the corresponding segments of the palmaris lateralis of the left and right forepaws of the rats. Parameters of EA stimulation were as follows: 2/100 Hz, intensity 1 mA, 30 min, and sparse‐dense wave. EA was initiated on day 7 after modeling from 9AM to 12AM once daily for 28 d, consecutively. Measurements of pain behavioral scores were obtained from 9AM to 12AM on days 7, 14, 21, 28, and 35. A schematic diagram of the experimental procedure was shown in Figure 1A.

### 2.4 Mechanical Withdrawal Threshold (MWT) and Thermal Withdrawal Latency (TWL) Test

The MWT and TWL test in each group were measured on days 0, 7, 14, 21, 28, and 35. Von Frey filaments with six different intensities (4, 6, 8, 10, 15, and 26 g) were chosen. First, a von Frey filament with minimum intensity (4 g) was vertically applied to the middle of the plantar surface of each paw until it bent, exerting a constant force for 2–5 s. A reaction was considered positive if the rat withdrew or licked its paws quickly. If there was no response, the next higher‐intensity von Frey filament was used until the rat produced a reaction that indicated the mechanical paw withdrawal threshold. Each testing session was separated by 5‐min interval. Using a thermal stimulator focused on the plantar surface of the hind paw, the thermal intensity was set at 50. The latency period was recorded when the rat quickly withdrew or licked its paw. The test was conducted in triplicate with a 5‐min interval between each repeat, and the average TWL of the hind paw was calculated.

### 2.5 Pain Behavioral Score

The pain‐related behavioral responses of the rats were observed before and after 10 min of EA stimulation. Pain behavior scores (S) were measured based on pain intensity, with behaviors ranked from low to high as follows: (1) licking of the abdomen or perineal area (L), (2) body stretching (B), (3) flattening of the abdomen against the floor (F), and (4) flanking contraction (C). The number of occurrences of each of these four behaviors within 10 min before and after EA stimulation was counted, and the pain score was calculated using the formula: S = 1 L + 2 B + 3 F + 4 C (L, B, F, and C represent the number of occurrences of each behavior within 10 min). The pain behavior score was evaluated using a single‐blind procedure, with higher scores indicating more severe pain. To calculate the adjusted pain behavior score (s'): s' = (S_After_/S_Before_) × S_Mean_. S_Before_ represents the pain behavior score within the first 10 min before EA stimulation, whereas S_After_ represents the pain behavior score within the first 10 min after EA stimulation. S_Mean_ represents the average pain behavior score of all rats with PID after 10 min of induction *E. coli* and *S. aureus*.

### 2.6 Gathering of Samples

Rats were anesthetized using isoflurane at the ending of the investigation. The serum, uterine tissue, dorsal root ganglia, local skin of sensitized acupoints (Guanyuan), and lumbosacral spinal cord tissues samples were collected and stored at −80°C.

### 2.7 Hematoxylin and Eosin (H&E) Staining

Among four groups, six rats in each group were used to make uterine tissue paraffin sections for H&E staining. The uterine tissues of rats were dehydrated, embedded, and cut into 5‐μm‐thick sections. Sections were dewaxed in water containing xylene and alcohol. They were stained with hematoxylin for 20 min and then submerged in warm water at 50°C until they turned blue. The sections were stained with eosin for 5 min and sealed with neutral gum. Images were acquired using a microscope (Motic China Group Co. Ltd., China), followed by histopathological damage scoring of the damaged uterine tissue. The scoring of uterine tissue damage was based on characteristic features, such as endometrial degeneration and necrosis, edema in the stromal layer, proliferative changes in the glandular layer, and inflammatory cell infiltration in the stromal and uterine muscle layers.

### 2.8 Enzyme‐Linked Immunosorbent Assay (ELISA)

The expressions of tumor necrosis factor‐α (TNF‐α, ZC‐37624), interleukin‐2 (IL‐2, ZC‐36393), transforming growth factor‐β1 (TGF‐β1, ZC‐37645), intercellular cell adhesion molecule‐1 (ICAM‐1, ZC‐37328), 5‐hydroxytryptamine receptor 3 (5‐HT3R, ZC‐35961), substance P (SP, ZC‐36170), hyaluronic acid (HA, ZC‐37271), bradykinin (BK, ZC‐36714) in serum samples and uterus tissues were measured by ELISA kits according to the manufacturer's instructions. All ELISA kits were purchased from ZCIBIO Technology Co. Ltd. (Shanghai, China). The microplate reader (SpectraMAX Plus384; Shanghai Molecular Devices Co. Ltd., China) was used to measure the absorbance (OD) at 450 nm.

### 2.9 Western Blot Analysis

Western blot analysis was used to measure the expression levels of 5‐HT3R, calcitonin gene‐related peptide (CGRP), hyaluronic acid (HA), kininogen 1 (KNG1), prostaglandin I2 (PGI2), and trefoil factor 2 (TFF2) in the skin of sensitized acupoints and plantar skin tissues. A bicinchoninic acid (BCA) kit (P0009; Beyotime, China) was used to assess protein concentrations. 10% sodium dodecyl sulfate‐polyacrylamide gel electrophoresis (SDS‐PAGE) was used to isolate the total protein. Following 2 h of transfer to polyvinylidene difluoride (PVDF) membranes at 200 mA for 2 h, the separated proteins were incubated for 2 h in 5% skim milk. The PVDF membranes were then treated with primary antibodies for a whole night at 4°C. The membranes were then treated with the secondary antibody for 90 min after being cleaned with phosphate‐buffered saline containing Tween‐20 (TBST). Electrochemiluminescence (ECL) was used to visualize the bands, and an internal control, β‐actin, was employed. The Tanon ECL gel imager (Shanghai, China) was used to capture pictures and scan the bands. The antibodies used were as follows: β‐actin (AC026, Abclonal, 1:50,000); 5‐HT3R (BS‐34076R, Bioss, 1:2,000); CGRP (A5542, Abclonal, 1:1,000); HA Tag (AF0039, Beyotime, 1:1,000); KNG1 (DF6544, Affinity, 1:2,000); PGI2 (ZY64571R, Shzeye, 1:2,000); TFF2 (66471‐1‐Ig, Proteintech, 1:1,000); HRP Goat Anti‐Mouse IgG (H + L) (AS003, Abclonal, 1:5,000); and Goat Anti‐Rabbit IgG (H + L) HRP (S0001, Affbiotech, 1:5,000).

### 2.10 Immunofluorescence (IF) Staining

Among four groups, six rats in each group were used to make dorsal root neuron tissue paraffin sections for IF staining. Paraffin‐embedded rat dorsal root neuron tissue sections were dewaxed and hydrated before incubation with 3% peroxide for 30 min at room temperature. Sections were heated in citrate buffer (pH 6) in a microwave for 20 min and washed three times with phosphate‐buffered saline (PBS) (5 min each). The sections were then exposed to primary antibodies (tyrosine hydroxylase (TH, ab129991, 1:100, Abcam) and neuronal nuclei (NeuN, ab177487, 1:100, Abcam)) overnight at 4°C, followed by washing three times with PBS. Next, CY3‐labeled goat anti‐rabbit IgG (GB21303, 1:100, Servicebio) and FITC‐labeled goat anti‐mouse IgG (GB22301, 1:100, Servicebio) were added and incubated at 37°C for 30 min. An OlyVIA fluorescence microscope (OLYMPUS, Tokyo, Japan) was used to observe staining, and ImageJ software (National Institutes of Health, USA) was employed to analyze the TH‐positive and NEUN‐positive area. Next, 4′,6‐diamidino‐2‐phenylindole (DAPI) staining was used to visualize the nuclei in blue, TH‐stained cells appeared green, and NeuN‐stained cells were red.

### 2.11 Transmission Electron Microscopy (TEM)

Samples were taken from lumbosacral spinal cord tissues of rats. TEM was used to observe the synaptic connections between the pelvic nerves of the dorsal commissural neuron (DCN), the afferent fiber terminals of the sciatic nerve, and projection neurons in the parabrachial nucleus (PBN). Samples were first exposed to 3% glutaraldehyde followed by 1% osmium tetroxide. Gradual dehydration was achieved using acetone. Next, ultrathin sections (60–90 nm) were prepared using an ultramicrotome. Sections were stained with uranyl acetate for 15 min, followed by lead citrate staining for 2 min. Finally, images were acquired using a TEM instrument (JEM‐1400FLASH, JEOL, Japan).

### 2.12 Data Analysis

SPSS software (version 17.0; SPSS Inc., Chicago, USA) was utilized for statistical analysis of the experimental findings, with data shown as mean ± standard deviation (SD). To compare multiple groups, a one‐way analysis of variance (ANOVA) was conducted. If the variances were equal, the LSD test was used; otherwise, Tamhane's T2 test was applied. Statistical significance was set at *p* < 0.05.

## 3 Results

### 3.1 Effects of EA at Guanyuan Acupoint at Different Frequencies on Pain Behavior of Rats With PID

To assess the effects of EA stimulation of the CV4 acupoint at different frequencies on the behavioral responses of rats in each group, we measured the behavioral pain scores of rats on days 7, 14, 21, 28, and 35. As shown in Figure 1B, the results of pain behavioral scores showed that compared with the control group, the pain behavioral scores of rats in the model group were significantly higher (all *p* < 0.01), but showed a decreasing trend over time. Compared with the model group, EA stimulation of CV4 at 2 Hz, 100 Hz, and 2/100 Hz significantly reduced the pain behavioral scores of rats (all *p* < 0.05) and EA stimulation of non‐sensitized acupoints did not have significant difference (*p* > 0.05). Interestingly, the group that received EA at 2/100 Hz exhibited a better analgesic effect than the other groups (all *p* < 0.01). Therefore, 2/100 Hz was selected for subsequent investigation. The pain behavioral scores consistently showed that EA stimulation of the CV4 at 2/100 Hz had a significant effect on reducing pain in rats with PID (Figure 1C). As shown in Figure 1D and E, MWT and TWL were significantly lower in the model group than in the control group (all *p* < 0.05), and MWT and TWL in rats with EA (2/100 Hz) stimulation of CV4 were significantly higher after day 14 compared to the model group (both *p* < 0.01), whereas EA stimulation of non‐sensitized acupoints was not statistically significant compared to the model group (*p* > 0.05). These results suggest that EA at CV4 increases the MWT and TWL in rats with PID and plays a pain‐relieving role.

### 3.2 EA at Guanyuan Acupoint Ameliorates the Uterus Histopathology of Rats With PID

**Figure 2:**
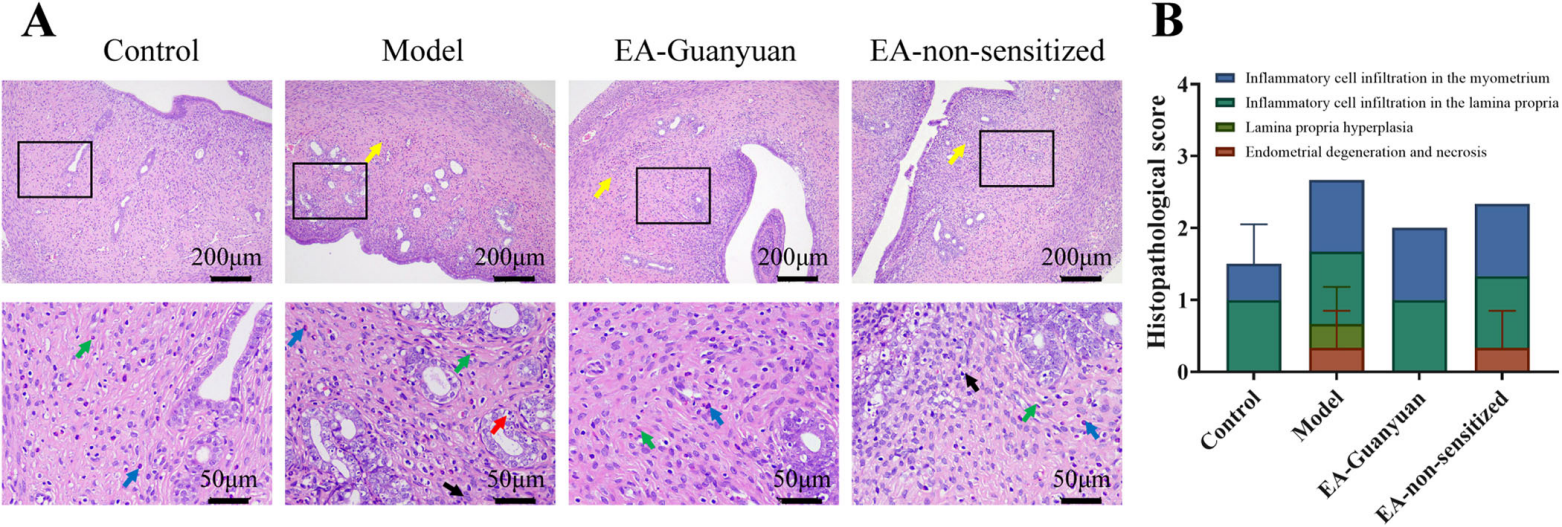
EA stimulation of Guanyuan acupoint ameliorates the uterus histopathology of rats with PID. Representative image of uterine tissue using H&E staining (A) and quantitative analysis of histopathological scores (B). Scale bars = 200 and 50 μm. The green arrow represents neutrophils, blue arrow represents eosinophils, red arrow represents degeneration and necrosis of glandular epithelial cells, black arrow represents fibroblasts, and yellow represents inflammatory cell infiltration.

As shown in Figure 2, histopathological analysis showed that the uterine tissue structure of normal rats was clear, the endometrial epithelium was a single layer of columnar epithelium arranged in a relatively regular manner, the uterine gland structure was normal, and glandular epithelial cells were neatly arranged. Compared to the control group, the model group showed degeneration and necrosis of endometrial epithelial cells, a small amount of degeneration and necrosis of stromal cells in the lamina propria, a small number of uterine gland hyperplasia, degeneration and necrosis of some glandular epithelial cells, and a large amount of inflammatory cell infiltration in the lamina propria and muscular layer. After treatment with EA stimulation at CV4, there was a certain improvement in the pathological changes of the uterine tissue; however, no significant difference was found (*p* > 0.05), and the treatment effect was more significant than that of the EA non‐sensitized acupoints. These results suggest that EA stimulation of CV4 attenuates uterine pathology in rats with PID.

### 3.3 EA at Guanyuan Acupoint Improves Inflammatory Response in Rats With PID

**Figure 3:**
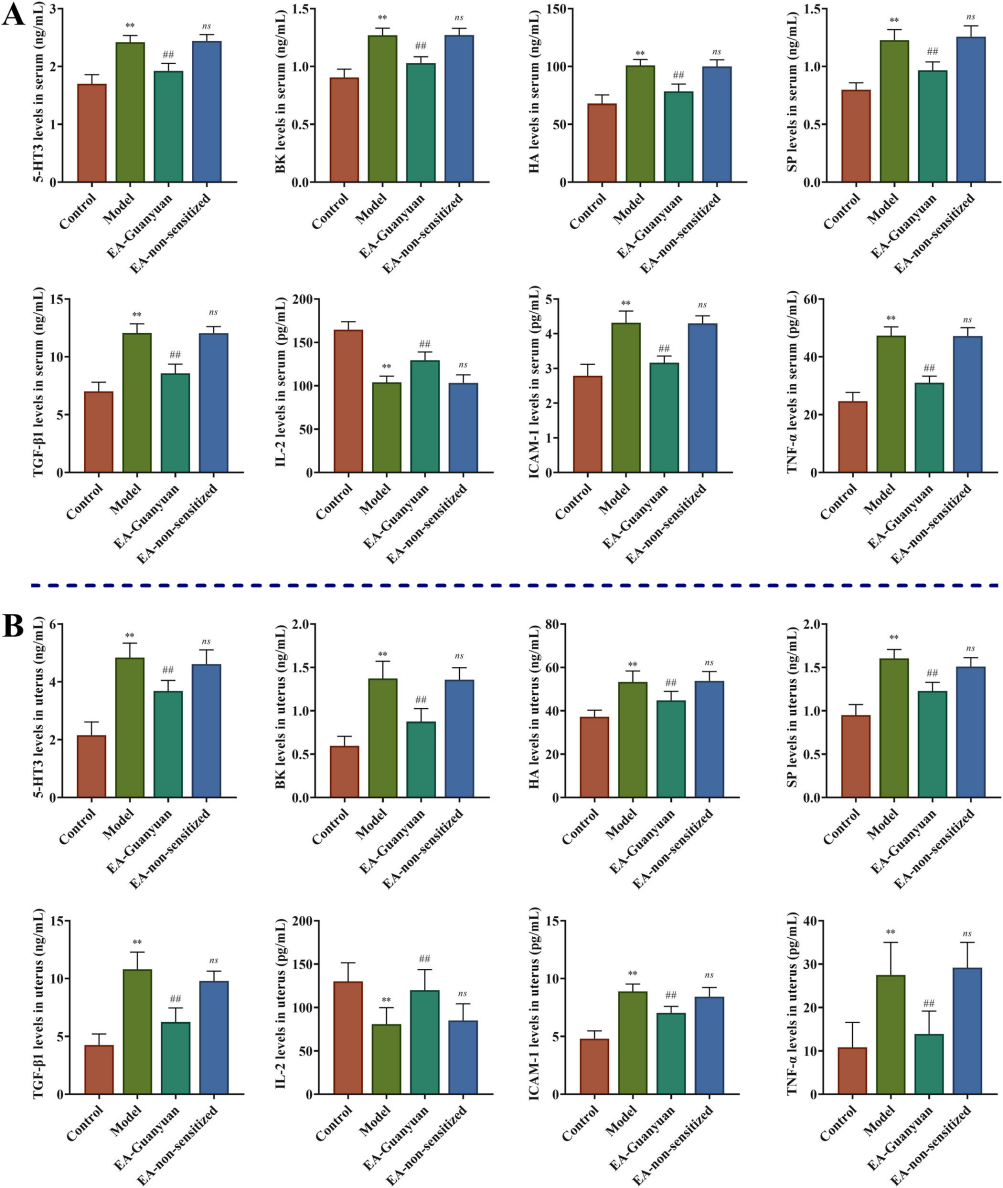
EA stimulation of Guanyuan acupoint improves inflammatory response in rats with PID. TNF‐α, IL‐2, TGF‐β1, ICAM‐1, 5‐HT3R, SP, HA, and BK expressions in serum samples (A) and uterus tissues (B) were measured using ELISA. Values are expressed as mean ± SD (*n* = 6), **p < 0.01, versus control group; ^##^ *p* < 0.01, ^*ns*^ *p* > 0.05, versus model group. TGF‐β1, transforming growth factor‐β1; ICAM‐1, intercellular cell adhesion molecule‐1; 5‐HT3R, 5‐hydroxytryptamine receptor 3; SP, substance P; HA, hyaluronic acid; BK, bradykinin.

To assess the effect of EA at CV4 on the inflammatory response in PID rats, the levels of inflammatory factors in serum samples and uterus tissues were detected using ELISA. As seen in Figure 3A and B, The levels of 5‐HT3R, BK, HA, SP, TGF‐β1, ICAM‐1, and TNF‐α expression in the serum and uterus of *E. coli‐* and *S. aureus*‐induced rats with PID was significantly higher than that of the control group, and IL‐2 levels were notably lower than that of the control group (all *p* < 0.01), whereas EA at CV4 reversed these changes (all *p* < 0.01). However, there was no significant difference between the EA‐non‐sensitized group and model group (all *p* > 0.05). These results suggest that EA stimulation of CV4 improves the inflammatory response in rats with PID by regulating the expression of inflammatory cytokines.

### 3.4 EA at Guanyuan Acupoint Reduces the Expression of Pain‐Causing Substances and Inflammatory Mediators in Rats With PID

**Figure 4:**
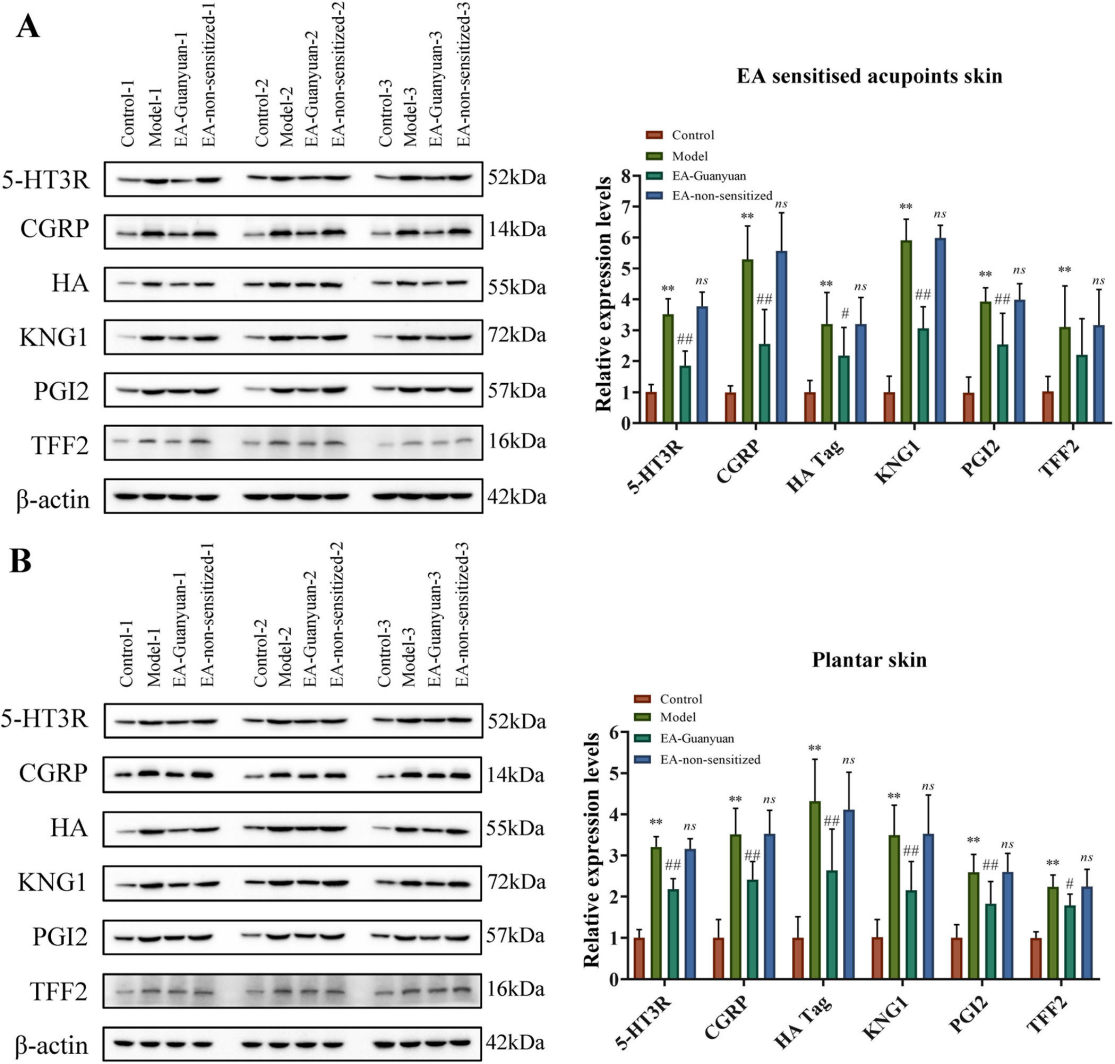
EA stimulation of Guanyuan acupoint reduces the expression of pain‐causing substances and inflammatory mediators in rats with PID. 5‐HT3R, CGRP, HA, KNG1, PGI2, and TFF2 expressions in EA stimulation of Guanyuan acupoint skin (A) and plantar skin (B) were detected using western blot analysis. Values are expressed as mean ± SD (*n* = 6), **p < 0.01, versus control group; ^#^ *p* < 0.05, ^##^ *p* < 0.01, ^*ns*^ *p* > 0.05, versus model group. 5‐HT3R, 5‐hydroxytryptamine receptor 3; CGRP, calcitonin‐gene‐related peptide; HA, hyaluronic acid; KNG1, Kininogen 1; PGI2, prostaglandin I2; TFF2, trefoil factor 2.

To investigate the mechanism by which EA stimulation of CV4 improves pain in rats with PID, the expression levels of neurogenic inflammatory response mediators and pain‐causing substances (5‐HT3R, CGRP, HA, KNG1, PGI2, and TFF2) were determined by western blot analysis. After EA stimulation of CV4 skin, the expression levels of 5‐HT3R, CGRP, HA, KNG1, and PGI2 were significantly increased in the model group compared to the control group (all *p* < 0.01) and were markedly reduced after treatment with EA stimulation of CV4 (all *p* < 0.05), except for TFF2, which showed no significant difference (Figure 4A). Furthermore, the same trend was observed in the plantar skin, consistent with the behavioral test sites (all *p* < 0.05) (Figure 4B). Briefly, EA stimulation of the CV4 relieved inflammatory pain in rats with PID by reducing the levels of inflammatory cytokines and pain‐causing substances.

### 3.5 EA at Guanyuan Acupoint Inhibits Sympathetic Activity and Restores the Synaptic Connections in Rats With PID

**Figure 5:**
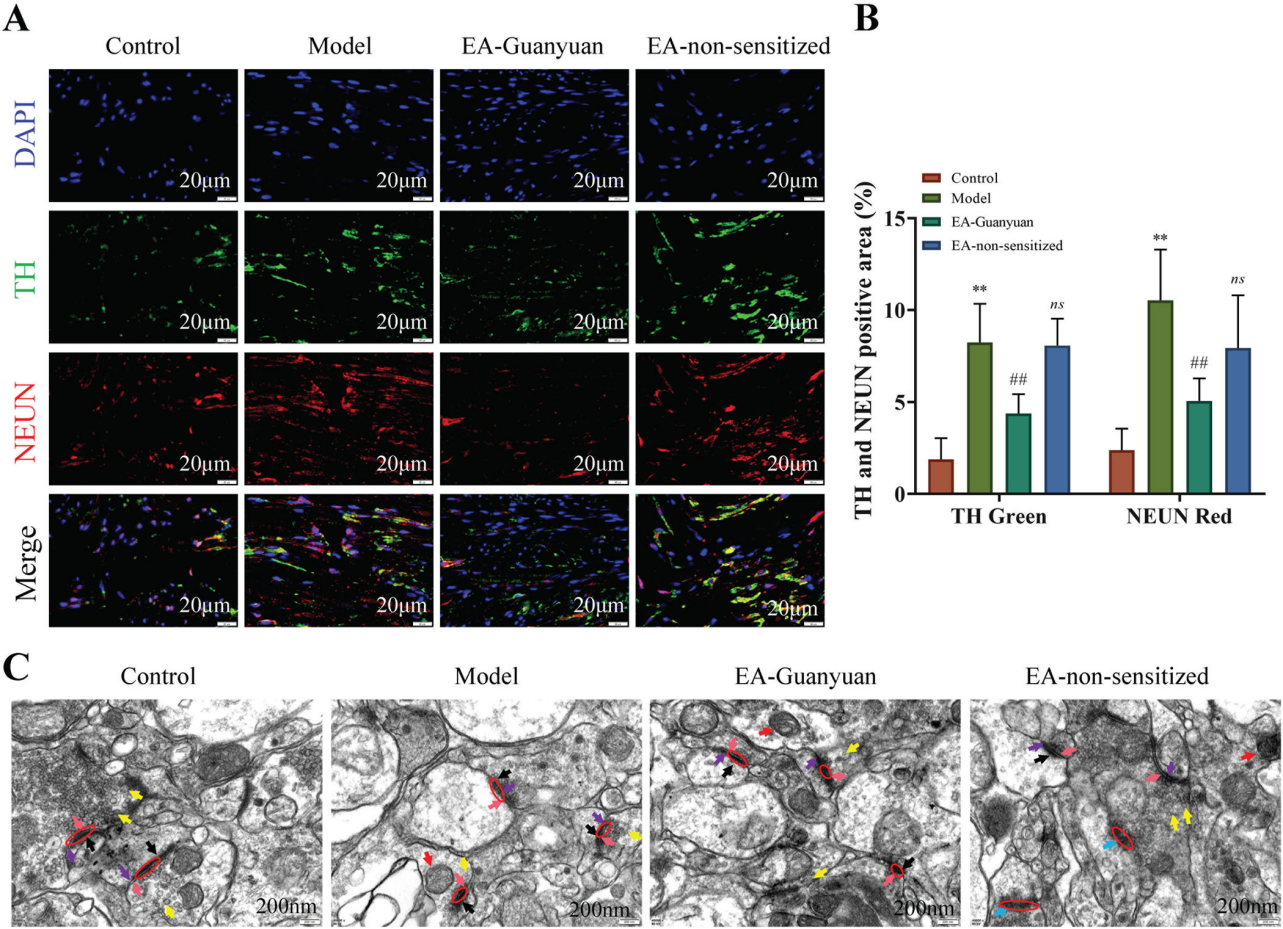
EA stimulation of Guanyuan acupoint inhibited sympathetic activity and restored the synaptic connections in rats with PID. Representative IF images of DAPI (blue), TH (green), and NeuN (red) staining in the dorsal root neuron tissue (A) and quantitative analysis of TH and NeuN positive area (B). Scale bar = 200 μm. (C) TEM was used to observe the synaptic connections between the pelvic nerves of DCN or the afferent fiber terminals of the sciatic nerve and projection neurons in the parabrachial nucleus. Mitochondrial condensation (red arrow), presynaptic membrane (pink arrow), postsynaptic membrane (purple arrow), postsynaptic membrane‐dense material (black arrow), synaptic vesicles (yellow arrow), difficulties in distinguishing presynaptic and postsynaptic membranes (blue arrow), and blurred synaptic cleft (circle) are shown. Scale bar = 200 nm. Values are expressed as mean ± SD (*n* = 6), **p < 0.01, versus control group; ^#^ *p* < 0.05, ^##^ *p* < 0.01, ^*ns*^ *p* > 0.05, versus model group. TH, tyrosine hydroxylase.

Sympathetic nerve marker TH was measured by IF staining. As shown in Figure 5A and B, TH and NeuN positive area was significantly elevated in the model group compared to the control group. Simultaneously, EA stimulation of CV4 cells reduced TH and NeuN positive area and inhibited sympathetic activity (Figure 5B). In addition, TEM was used to observe the synaptic connections between the pelvic nerves of the DCN, the afferent fiber terminals of the sciatic nerve, and projection neurons in the parabrachial nucleus. As shown in Figure 5C, in the control group, the synaptic structure was intact, the presynaptic membrane, postsynaptic membrane, and synaptic gap were visible, and the synaptic gap was clear. Compared to the control group, the model group mitochondria solidified, the contact area of the presynaptic and postsynaptic membranes became smaller, the synaptic gap was blurred, the dense substance of the postsynaptic membrane was slightly thickened, and the synaptic vesicles were reduced. Synaptic contacts were enhanced after EA treatment. These results indicated that EA stimulation of CV4 ameliorated pelvic inflammatory pain, possibly by inhibiting sympathetic activity and modulating the connection between the sympathetic and sensory nerves through synapses.

## 4 Discussion

PID is a common gynecological disease with a high recurrence rate. In recent years, its annual incidence has increased, causing extreme pain in most female patients. TCM theory provides several methods for treating pelvic inflammation, among which acupuncture can exert the characteristics and advantages of Chinese medicine. The Guanyuan acupoint (CV4), located three inches below the navel, is the intersection of the Ren Meridian and Foot‐Threeyin Meridians, which reinforces the vital essence and strengthens the primordial qi [24]. Evidence from clinical studies supported the high efficacy of CV4 acupuncture in managing gynecological ailments [25]. The combination of CV4 with Sanyinjiao (SP6) and Zigong (EX‐CA1) has been demonstrated to considerably reduced the symptoms in patients with chronic pelvic inflammation [26]. However, there is limited research on the molecular mechanisms through which acupuncture at the CV4 improves pelvic inflammation.

Sensitization of acupoints refers to a biological process in which an organism undergoes a neurogenic inflammatory reaction as the primary characteristic and uses inflammatory mediators as endogenous regulatory factors under pathological conditions [27]. When the viscera are stimulated, corresponding acupoints on the body surface may exhibit abnormal sensitization of pain perception, resulting in a transition from a “resting state” to an “activated state.” When there is damage to the visceral organs, it often leads to pain or hyperalgesia, which is also referred to as somatic referred pain [28, 29]. The area of the body surface where pain reflects the corresponding sensitization point [30]. There is evidence that the locations of these sensitized points overlap significantly with the corresponding acupuncture points [31, 32]. In recent years, research has shown that EA stimulation of sensitized acupoints can alleviate visceral pain [33, 34]. Therefore, we selected CV4 as the sensitized acupoint and the corresponding segments of the left and right forepaws as the non‐sensitized acupoints in this study. In our study, it was confirmed that EA stimulation of CV4 reduced the pain threshold and pain score of rats and ameliorated pathological damage to the uterine tissue.

PID is primarily caused by chronic inflammatory infiltration, leading to fibrous connective tissue proliferation, adhesion, and contraction, thereby causing nerve fiber compression and pain [35]. Inflammatory responses play an important role in PID pathogenesis. Damage to the body by external stimuli leads to the release of numerous inflammatory factors and pain‐inducing substances that cause visceral neurogenic inflammatory responses and hyperalgesia [36]. 5‐HT3R is a 5‐HT receptor that participates in the regulation of neuronal development [37]. Evidence suggests that CGRP plays a critical role in pain mechanisms [38]. SP extensively found in both the central and peripheral nervous systems and is involved in the pathophysiological processes of pain and inflammatory diseases [39]. HA is a major component of extracellular and plays a crucial role in the regulation of inflammation [40]. BK is the major peptide in the kallikrein‐kinin system, and its activation can induce neuronal excitation and is closely related to neural differentiation, inflammation, and pain hypersensitivity [41, 42]. PGI2, the final product of arachidonic acid metabolism, plays a key role in neuropathic pain [43]. We looked into the expression of pain‐causing substances and inflammatory factors connected to neurogenic inflammation in this study. These results confirmed that EA stimulation at CV4 reduced the expression of inflammatory factors and pain‐causing substances, improving the neurogenic inflammatory response and referred pain in rats with PID.

In addition, when the viscera is injured, signals are primarily transmitted to the CNS through incoming fibers that accompany the sympathetic nerves. Pain perception and inhibition occur with adjustments at various levels of the CNS [44]. Spinal dorsal horn neurons are activated in the forward direction through the transmission of nociceptive information from the viscera through dorsal root ganglion (DRG) neurons. After establishing synapses with secondary neurons, electrical nerve signals are transmitted in the reverse direction to peripheral nerves, resulting in the sensation of pain sensitivity [45]. In addition, under pathological conditions, the excitability of DRG neurons increases abnormally, leading to the release of neuropeptides and inflammatory factors [46]. Furthermore, studies have found that after excitation of the sympathetic nervous system, which causes pain hypersensitivity, it can generate sympathetic nerve sprouts that surround sensory neurons and establish multiple synaptic contacts with damaged incoming nerve axons [47, 48]. The present study indicated that rats exhibited sympathetic nerve excitation in response to stimulation by pathogenic bacteria, leading to the prolonged presence of local inflammatory and painful substances around the nociceptors. This is manifested as an elevation in TH expression, which in turn causes pain sensitization. Our study showed that EA at CV4 reduced TH positive expression area in rats with PID. In addition, we demonstrated that primary input information from the pelvic viscera can be transmitted to PBN‐projecting neurons in the DCN through synapses, indicating a close relationship between the DCN and transmission of pelvic visceral pain information. Studies have shown that Spinal dorsal horn neurons are involved in the central amplification mechanism of visceral organ sensation [49]. We speculate that these neurons, upon receiving primary input from various sources, may activate relevant signaling pathways and participate in pain perception; however, the specific mechanisms require further investigation. In addition, we admit that our research still has some shortcomings. This study was based on a nonclinical sample, and we will continue working on in vivo testing to reveal potential clinical applications of the Guanyuan acupoint in our future studies, including its safety and efficacy.

## 5 Conclusions

In summary, this study demonstrated that EA at CV4 could alleviate the pathological damage and pain sensitization of the uterine tissue in rats with PID by inhibiting sympathetic activity and neurogenic inflammatory response. This study contributes to the elucidation of the mechanism and biological basis of acupoint sensitization from a neurophysiological perspective and provides a scientific basis for understanding the molecular mechanism of EA at sensitized acupoints in PID treatment.

## Author Contributions

**Jinyu Qu:** conceptualization, methodology, writing – original draft. **Yingchun Peng:** conceptualization, investigation, methodology, writing – original draft. **Xuefang Shen:** data curation, formal analysis. **Jin Xiong:** data curation, formal analysis. **Huan Wang:** data curation, formal analysis. **Xiang Xiao:** writing – review and editing. **Yili Wang:** conceptualization, funding acquisition, methodology, supervision, writing – review and editing.

## Ethics Statement

The procedures in this experiment were performed in accordance with protocols approved by the Experimental Animal Welfare Ethics Committee of Chengdu Medical College (Approval No. 2023012).

## Consent

The authors have nothing to report.

## Conflict of Interests

The authors declare no conflicts of interest.

## Data Availability

The datasets used and/or analyzed during the current study are available from the corresponding author on reasonable request.
